# Mobile App Rating Scale: A New Tool for Assessing the Quality of Health Mobile Apps

**DOI:** 10.2196/mhealth.3422

**Published:** 2015-03-11

**Authors:** Stoyan R Stoyanov, Leanne Hides, David J Kavanagh, Oksana Zelenko, Dian Tjondronegoro, Madhavan Mani

**Affiliations:** ^1^Institute of Health & Biomedical InnovationSchool of Psychology and CounsellingQueensland University of Technology (QUT)Brisbane, QLDAustralia; ^2^The Young and Well Cooperative Research Centre (Young and Well CRC)Abbotsford, VICAustralia; ^3^School of DesignCreative Industries FacultyQueensland University of Technology (QUT)Brisbane, QLDAustralia; ^4^Information SystemsScience and Engineering FacultyQueensland University of Technology (QUT)Brisbane, QLDAustralia

**Keywords:** well being, mental health, e-health, mobile health (mhealth), mobile application, assessment, rating, scale development

## Abstract

**Background:**

The use of mobile apps for health and well being promotion has grown exponentially in recent years. Yet, there is currently no app-quality assessment tool beyond “star”-ratings.

**Objective:**

The objective of this study was to develop a reliable, multidimensional measure for trialling, classifying, and rating the quality of mobile health apps.

**Methods:**

A literature search was conducted to identify articles containing explicit Web or app quality rating criteria published between January 2000 and January 2013. Existing criteria for the assessment of app quality were categorized by an expert panel to develop the new Mobile App Rating Scale (MARS) subscales, items, descriptors, and anchors. There were sixty well being apps that were randomly selected using an iTunes search for MARS rating. There were ten that were used to pilot the rating procedure, and the remaining 50 provided data on interrater reliability.

**Results:**

There were 372 explicit criteria for assessing Web or app quality that were extracted from 25 published papers, conference proceedings, and Internet resources. There were five broad categories of criteria that were identified including four objective quality scales: engagement, functionality, aesthetics, and information quality; and one subjective quality scale; which were refined into the 23-item MARS. The MARS demonstrated excellent internal consistency (alpha = .90) and interrater reliability intraclass correlation coefficient (ICC = .79).

**Conclusions:**

The MARS is a simple, objective, and reliable tool for classifying and assessing the quality of mobile health apps. It can also be used to provide a checklist for the design and development of new high quality health apps.

##  Introduction

### Global Smart Phone App Usage

The use of mobile apps for health and well being promotion has grown exponentially in recent years [1]. Between 2013 and 2014 the global use of smart phones increased by 406 million, reaching 1.82 billion devices (up 5% in a year), and Internet usage via mobile devices has increased by 81% in one year [2]. There were 13.4 billion apps that were downloaded in the first quarter of 2013 [3], with projected figures of 102 billion for the whole year [4]. The portability of smart phones provides access to health information and interventions at any time in any context. The capabilities (eg, sensors) of smart phones can also enhance the delivery of these health resources.

Given the rapid proliferation of smart phone apps, it is increasingly difficult for users, health professionals, and researchers to readily identify and assess high quality apps [5]. Little information on the quality of apps is available, beyond the star ratings published on retailers’ Web pages, and app reviews are subjective by nature and may come from suspicious sources [6]. Selecting apps on the basis of popularity yields little or no meaningful information on app quality [7].

Much of the published literature focuses on technical aspects of websites, presented mostly in the form of checklists, which do not assess the quality of these features [8-10]. Website quality can be described as a function of: (1) *content*, (2) *appearance and multimedia*, (3) *navigation*, (4) *structure and design*, and (5) *uniqueness* [11]. A synthesis of website evaluation criteria conducted by Kim et al [12] shortlisted 165 evaluation criteria, grouped in 13 groups (eg, *design and aesthetics, ease of use*). However, 33 criteria were unable to be grouped and were coded as *“*miscellaneous*”*, highlighting the complexity of the task. While many website criteria may be applicable to mobile apps, there is a need to consider whether a specific quality rating scale may be needed for apps.

Attempts to develop mobile health (mHealth) evaluation criteria are often too general, complex, or specific to a particular health domain. Handel [13] reviewed 35 health and well being mobile apps based on user ratings of: (1) *ease of use*, (2) *reliability*, (3) *quality*, (4) *scope of information*, and (5) *aesthetics.* While these criteria may cover important aspects of quality, no rationale for these specific criteria was provided. Khoja et al [14] described the development of a matrix of evaluation criteria, divided into seven themes for each of the four stages of an app’s life-cycle: (1) *development*, (2) *implementation*, (3) *integration*, and (4) *sustained operation*. While this matrix provides comprehensive criteria for rating app quality, the complex and time-consuming nature of the evaluation scheme would be difficult to apply in routine practice and research. Furthermore, the matrix omits any evaluation of the visual *aesthetics* of the app as a criterion.

Guidelines for evaluating the usability of mHealth apps were also compiled by the Health Care Information and Management Systems Society (HIMSS) [15]. These guidelines use a “Strongly agree” to “Strongly disagree” Likert scale to rate each criterion, which does not provide an indication of their quality. Strong agreement that a criterion is met (ie, clarity in whether a feature is present) is not necessarily equivalent to meeting the criterion to a high degree. While the HIMSS criteria were extensive, and included usability criteria for rating *efficiency*, *effectiveness*, *user satisfaction*, and *platform optimization*, no criteria for rating *information quality* were included. This is problematic, as failure to evaluate the accuracy and appropriateness of the health information contained in mHealth apps could compromise user health and safety [16].

A reliable and objective instrument is needed to rate the degree that mHealth apps satisfy quality criteria. This scale should be easy to understand and use with minimal training. This scale will initially be used by researchers, but may later be made available to app developers and health professionals, pending further research.

### Objectives

The objective of this study is to develop a reliable, multidimensional scale for classifying and rating the quality of mobile health apps.

##  Methods

### Mobile App Rating Scale Development

A comprehensive literature search was conducted to identify articles containing explicit Web- or app-related quality rating criteria. English-language papers from January 2000 through January 2013 were retrieved from PsycINFO, ProQuest, EBSCOhost, IEEE Xplore, Web of Science, and ScienceDirect. The search terms were, “mobile” AND “app*” OR “web*” PAIRED WITH “quality” OR “criteria” OR “assess*” OR “evaluat*”.

Three key websites, including the EU’s Usability Sciences [17], Nielsen Norman Group’s user experience (UX) criteria, and HIMSS were searched for relevant information. References of retrieved articles were also hand-searched. Professional research manuals, unpublished manuscripts, and conference proceedings were also explored for additional quality criteria. After initial screening of title and abstract, only studies that reported quality assessment criteria for apps or Web content were included.

Website and app assessment criteria identified in previous research were extracted. Criteria irrelevant to mobile content and duplicates were removed. An advisory team of psychologists, interaction and interface designers and developers, and professionals involved in the development of mHealth apps worked together to classify assessment criteria into categories and subcategories, and develop the scale items and descriptors. Additional items assessing the app’s description in the Internet store and its evidence base were added. Corrections were made until agreement between all panel members was reached.

### Mobile App Rating Scale Testing on Mental Health Apps

A systematic search of the Apple iTunes store was conducted on September 19, 2013, following the PRISMA guidelines for systematic literature reviews [18]. An exhaustive list of mental-health related mobile apps was created. The following search terms were employed, “Mindfulness” OR “Depression” OR “Wellbeing” OR “Well-being” OR “Mental Health” OR “Anger” OR “CBT” OR “Stress” OR “Distress” OR “Anxiety”.

App inclusion criteria were: (1) English language; (2) free of charge; (3) availability in the Australian iTunes store; and (4) from iTunes categories, “Health & Fitness”, “Lifestyle”, “Medical”, “Productivity”, “Music”, “Education”, and “Utilities”. The category inclusion criteria were based on careful scrutiny of the titles and types of apps present in those categories.

There were 60 apps that were randomly selected using a randomization website [19]. The first ten were used for training and piloting purposes. There were two expert raters: (1) a research officer with a Research Masters in Psychology and two years’ experience in mobile app development, and (2) a PhD candidate with a Masters degree in Applied Psychology and over nine years information technology experience, that trialled each of the first 10 apps for a minimum of 10 minutes and then independently rated their quality using the Mobile App Rating Scale (MARS). The raters convened to compare ratings and address ambiguities in the scale content until consensus was reached. The MARS was revised based on that experience, and the remaining 50 mental health and well being related apps were trialled and independently rated. A minimum sample size of 41 is required to establish whether the true interrater reliability lies within .15 of a sample observation of .80, with 87% assurance (based on 10,000 simulation runs) [20]. The sample size of 50, therefore, provides substantial confidence in the estimation of the interrater reliability in the current study. Data were analyzed with SPSS version 21 (SPSS Inc, Chicago, IL, USA). The internal consistency of the MARS quality subscales and total quality score was calculated using Cronbach alpha. This indicates the degree (correlations) to which items measuring the same general construct produce similar scores. Interrater reliability of the MARS subscales and total score was determined by the intraclass correlation coefficient (ICC) [21]. This statistic allows for the appropriate calculation of weighted values of rater agreement and accounts for proximity, rather than equality of ratings. A two-way mixed effects, average measures model with absolute agreement was utilized [22]. The concurrent validity of the MARS total score was examined in relation to the Apple iTunes App Store average star rating for each app (collected from the Apple iTunes App Store on September 19, 2013).

## Results

### Mobile App Rating Scale Development

The search strategy yielded 25 publications, including peer-reviewed journal articles (n=14), conference proceedings (n=8), and Internet resources (n=3) containing explicit mobile or Web-related quality criteria. The complete list of utilized resources is available with this article (see Multimedia Appendix 1, papers, publications, and materials used for MARS criteria selection). A total of 427 criteria were extracted, 56 were removed as duplicates, and 22 were deemed irrelevant to apps. The remaining 349 criteria were grouped into six categories by the expert panel, one relating to app *classification*, four categories on objective app qualities (*engagement*, *functionality*, *aesthetics,* and *information quality*), and one on *subjective app quality* (see Table 1), through an iterative approach.

**Table 1:** Number of criteria for evaluation of mHealth app quality identified in the literature search.

Criterion category	Frequency, N=349	(%)
App classification, confidentiality, security, registration, community, affiliation	12	(3.4)
Aesthetics, graphics, layout, visual appeal	52	(14.8)
Engagement, entertainment, customization, interactivity, fit to target group, etc	66	(18.9)
Functionality, performance, navigation, gestural design, ease of use	90	(25.8)
Information, quality, quantity, visual information, credibility, goals, description	113	(32.4)
Subjective quality, worth recommending, stimulates repeat use, overall satisfaction rating	16	(4.6)

### Classification Category

The *classification* category collected descriptive information on the app (eg, price, platform, rating) as well as its technical aspects (eg, log-in, password-protection, sharing capabilities). Additional sections collect information on the target age group of the app (if relevant), as well as information on what aspects of health (including physical health, mental health, well-being) the app targets. These domains may be adapted to include/exclude specific content areas as needed.

The app quality criteria were clustered within the *engagement*, *functionality*, *aesthetics*, *information quality,* and *subjective quality* categories, to develop 23 subcategories from which the 23 individual MARS items were developed. Each MARS item used a 5-point scale (1-Inadequate, 2-Poor, 3-Acceptable, 4-Good, 5-Excellent), descriptors for these rating anchors were written for each item. In cases where an item may not be applicable for all apps, an option of Not applicable was included. The expert panel scrutinized the MARS items and rating descriptor terminology to ensure appropriate and consistent language was used throughout the scale.

Calculating the mean scores of the e*ngagement*, *functionality*, *aesthetics*, and *information quality* objective subscales, and an overall mean app quality total score is how the MARS is scored. Mean scores instead of total scores are used because an item can be rated as Not applicable. Additionally, mean scores are used to provide quality ratings corresponding to the familiar format of star ratings. The *subjective quality* items can be scored separately as individual items, or a mean *subjective quality* score. The MARS *app classification* section is for descriptive purposes only.

### Mobile App Rating Scale Testing

**Figure 1:**
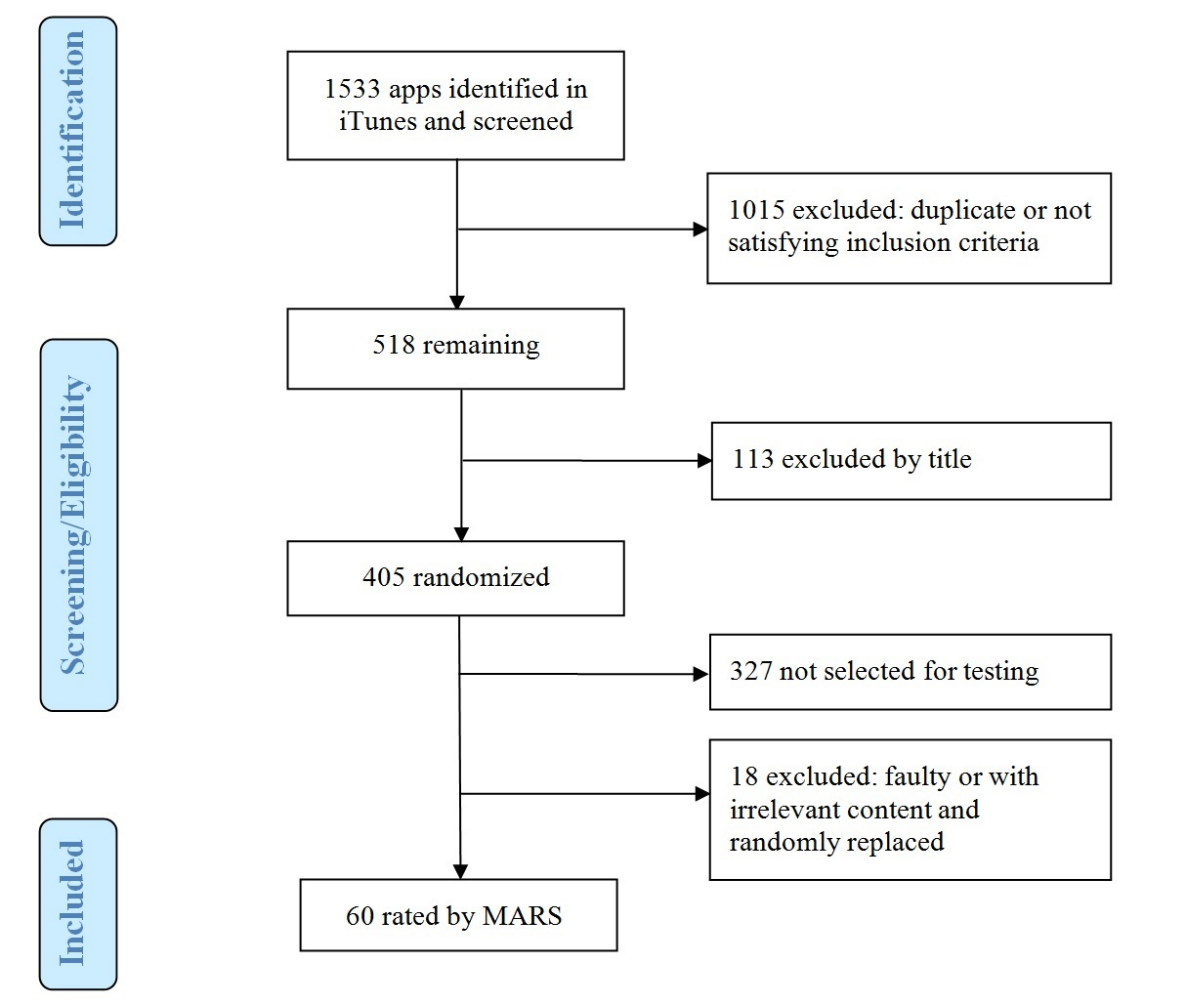
Flow diagram of the process utilized to identify apps for piloting the Mobile App Rating Scale (MARS).

A total of 1533 apps were retrieved from the iTunes search. All duplicate, non-English, and paid apps were removed. Apps from the categories “games”; “books”; “business”; “catalog”; “entertainment”; “finance”; “navigation”; “news”; “social networking”; and “travel” were also removed. Remaining apps were screened by title. The app store descriptions of apps with unclear titles were reviewed prior to exclusion. App titles with the words “magazine”, “mother”, “mum”, “job”, “festival”, “massage”, “shop”, or “conference”, as well as company ads and Web apps were also excluded, as they were linked to irrelevant content. There were sixty of the remaining 405 apps that were randomly selected for rating with the MARS (Figure 1 shows this).

On attempting to rate the initial ten apps, it was found that one was faulty and could not be rated. MARS ratings of the remaining nine apps indicated the scale had a high level of internal consistency (Cronbach alpha = .78) and fair interrater reliability (2-way mixed ICC = .57, 95% CI 0.41-0.69). The Not applicable option was removed from items within the *engagement* category, as this feature was considered to be an important and universal component of all high-quality apps. The meaning of *visual information* was clarified and the item rephrased. The stated or inferred age target of “young people” was defined as app users age 16-25. The descriptor of *goals* was clarified to read; *Does the app have specific, measurable, and achievable goals (specified in app store description or within the app itself)?*; to help distinguish it from the item *accuracy of app description*, which often relates to the app’s goals. On the *information* subscale, raters found it difficult to determine when lack of information within an app should be rated as Not applicable or as a flaw; this item was therefore revised to require that information be rated unless the apps were purely for entertainment. The final version of the MARS is provided with this article (see Multimedia Appendix 2, Mobile App Rating Scale).

Independent ratings on the overall MARS total score of the remaining 50 mental health and well being apps demonstrated an excellent level of interrater reliability (2-way mixed ICC = .79, 95% CI 0.75-0.83). The MARS total score had excellent internal consistency (Cronbach alpha = .90) and was highly correlated with the MARS star rating item (#23), *r*(50) = .89, *P*<.001. Internal consistencies of the MARS subscales were also very high (Cronbach alpha = .80-.89, median .85), and their interrater reliabilities were fair to excellent (ICC = .50-.80, median .65). Detailed item and subscale statistics are presented in Table 2. A full list of the apps, which were trialled and rated, using the MARS, as well as their mean objective and subjective app quality scores is provided with this article (see Multimedia Appendix 3, Mobile Apps Used for MARS Evaluation).

Only 15 of the 50 mental health and well being apps extracted from the iTunes App Store had received the five user ratings required for a star rating to be displayed. These apps showed a moderate correlation between the iTunes star rating and the total MARS score (*r*(15) = .55, *P*<.05).

**Table 2:** Interrater reliability and internal consistency of the MARS items and subscale scores, and corrected item-total correlations and descriptive statistics of items, based on independent ratings of 50 mental health and well being apps.

#		Subscale/item	Corrected item-total correlation		Mean	SD
**Engagement alpha = 0.89, ICC = 0.80 (95% CI 0.73-0.85)**						
	1	Entertainment		.63	2.49	1.24
	2	Interest		.69	2.52	1.20
	3	Customization		.60	2.27	1.15
	4	Interactivity		.65	2.70	1.22
	5	Target group		.61	3.41	0.93
**Functionality alpha = 0.80, ICC = 0.50 (95% CI 0.33-0.62)**						
	6	Performance		.42	4.00	0.93
	7	Ease of use		.29	3.93	0.87
	8	Navigation		.48	4.00	0.94
	9	Gestural design		.48	4.10	0.79
**Aesthetics alpha = 0.86, ICC = 0.61 (95% CI 0.46-0.72)**						
	10	Layout		.56	3.91	0.87
	11	Graphics		.61	3.41	0.92
	12	Visual appeal: How good does the app look?		.60	3.14	0.91
**Information alpha = 0.81, ICC = 0.79 (95% CI 0.71-0.84)**						
	13	Accuracy of app description		.67	3.66	1.03
	14	Goals		.70	3.43	1.10
	15	Quality of information		.47	3.18	1.46
	16	Quantity of information		.58	2.87	1.54
	17	Visual information		.39	1.35	1.89
	18	Credibility		.46	2.79	0.95
	19	Evidence base^a^		-	-	-
**Subjective quality alpha = 0.93, ICC = 0.83 (95% CI 0.75-0.88)** ^b^						
	20	Would you recommend this app?		.84	2.31	1.17
	21	How many times do you think you would use this app?		.82	2.46	1.12
	22	Would you pay for this app?		.63	1.31	0.60
	23	What is your overall star rating of the app?		.89	2.69	1.06

^a^ Item 19 “Evidence base” was excluded from all calculations, as it currently contains no measurable data.

^b^ The *Subjective quality* subscale was excluded from the total MARS ICC calculation.

##  Discussion

### Principal Results

The MARS is the first mHealth app quality rating tool, to our knowledge, to provide a multidimensional measure of the app quality indicators of *engagement, functionality, aesthetics*, and *information quality,* as well as app *subjective quality*. These app quality indicators were extracted from previous research across the UX, technical, human-computer interaction, and mHealth literature, but had not previously been combined in a singular framework. Previous attempts to develop mobile app evaluation criteria have been too technical or specific to a particular health domain. They have also not been developed and piloted in a systematic manner using an expert panel of health professionals, designers, and developers of health Web and mobile apps. In contrast, the MARS is an easy-to-use (with appropriate training), simple, objective, reliable, and widely applicable measure of app quality, developed by an expert multidisciplinary team. Although the generalizability of the MARS is yet to be tested, the scale can be modified to measure the quality of nonhealth related apps. The MARS total mean score describes the overall quality of an app, while the mean *engagement*, *functionality*, *aesthetics*, and *information quality* subscale scores can be used to describe its specific strengths and weaknesses.

The use of objective MARS item anchors and the high level of interrater reliability obtained in the current study should allow health practitioners and researchers to use the scale with confidence. Both the app quality total score and four app-quality subscales had high internal consistency, indicating that the MARS provides raters with a reliable indicator of overall app quality, as well as the quality of app *engagement*, *functionality*, *aesthetics*, and *information quality*. The exclusion of the *subjective quality* subscale from the overall mean app quality score, due to its subjective nature, strengthens the objectivity of the MARS as a measure of app quality. Nevertheless, the high correlation between the MARS quality total score and its overall star rating provides a further indication that it is capturing perceived overall quality. It should be noted that the MARS overall star rating is likely to be influenced by the prior completion of the 19 MARS app quality items. Nevertheless, the iTunes App Store star ratings available on 15 of the 50 mental health apps rated were only moderately correlated with the MARS total score. This was unsurprising; given the variable criteria likely to be used by different raters, the subjective nature of these ratings, and the lack of reliability of the iTunes star ratings, as has been highlighted in previous research [6]. In addition, the MARS overall star rating score was only moderately correlated with the iTunes App Store star rating. The MARS star rating is likely to provide a more reliable measure of overall app quality, as it is rated following completion of the entire MARS, and is therefore informed by the preceding items.

It is recommended that MARS raters complete a training exercise before commencing use. Training slides are available from the corresponding author. If multiple MARS raters are utilized, it is recommended that raters develop a shared understanding of the target group for the apps, clarify the meaning of any MARS items they find ambiguous, and determine if all MARS items and subscales are relevant to the specific health area of interest. App-quality ratings should be piloted and reviewed until an appropriate level of interrater reliability or consensus ratings are reached. The MARS also assumes that raters have undertaken a detailed exploration of the app’s content and functionalities.

Due to the generic nature of the mHealth app quality indicators included in the MARS, it is recommended that a number of “App-Specific” items are added to obtain information on the perceived impact of the app on the user’s knowledge, attitudes, and intentions related to the target health behavior (see *App Specific* section of the MARS).

For convenience, the MARS was piloted on iPhone, rather than Android apps. Since initial testing, however, the scale has been applied to multiple Android apps and no compatibility issues were encountered. However, future research should explore the reliability of the scale with Android apps.

### Limitations

While the original search strategy to identify app-quality rating criteria was conducted using guidelines for a systematic review, few peer-reviewed journal articles were identified. As a result, the search strategy was expanded to include conference proceedings and Internet resources, which may not have been as extensively peer reviewed. Suggested guidelines for scale-development were followed [23], whereby a qualitative analysis of existing research was conducted to extract app-quality criteria and then develop app-quality categories, subcategories, MARS items, and their anchor ratings via a thematic review and expert panel ratings. Despite these efforts, and the corrections made after piloting the scale, two MARS items on the *functionality* subscale (*ease of use* and *navigation*) achieved only moderate levels of interrater reliability (ICC = .50). These items have been revised and are being tested.

Researchers are yet to test the impact of the mental health apps included in this study. As a result, the MARS item *evidence base* was not rated for any of the apps in the current study and its performance has not been tested. It is hoped that as the evidence base for health apps develops, the applicability of this MARS item will be tested.

### Future Research

Future research is required to determine the suitability and reliability of the MARS across multiple health and other app domains, as well as its applicability in the sphere of app development. The association of the app quality total and subscale scores with the concepts of user experience, quality of experience, and quality of service requires further investigation. Future refinements of MARS terminology and additional items are likely to be required, as the functionality of mobile apps progresses. It is hoped the current version of the MARS provides mHealth app-developers with a checklist of criteria for ensuring the design of high-quality apps.

The MARS could also be utilized to provide quantitative information on the quality of medical apps as part of recent medical app peer-review initiatives, such as that launched by JMIR mHealth and uHealth [24].

With some modification, the MARS may also inform the development and quality rating of health-related websites. While the MARS was designed to be utilized by experts in the mHealth field, a simpler version of the scale, “MARS-app user”, based on the original MARS, was developed in consultation with youth agencies and young people for the purposes of obtaining user feedback on app quality and satisfaction. The MARS-app user version is currently being piloted. It is available upon request from the corresponding author.

Future research is also required to determine how to best evaluate the safety of mHealth apps in terms of the quality of the health information contained in the apps and the privacy and security of user information [16,25]. Su [25] recently suggested that assessment of the security and integrity of mHealth apps should include exploration of open-source developer codes for potential malicious functions.

### Conclusions

The MARS provides a multidimensional, reliable, and flexible app-quality rating scale for researchers, developers, and health-professionals. Current results suggest that the MARS is a reliable measure of health app quality, provided raters are sufficiently and appropriately trained.

## Multimedia Appendix 1

Papers, publications, and materials used for MARS criteria selection.

## Multimedia Appendix 2

Mobile App Rating Scale.

## Multimedia Appendix 3

Mobile Apps Used for MARS Evaluation.

## Abbreviations

CBT: cognitive behavioral therapy
HIMSS: Health Care Information and Management Systems Society
ICC: intraclass correlation coefficient
MARS: Mobile App Rating Scale
mHealth: mobile health
UX: user experience
Young and Well CRC: Young and Well Cooperative Research Centre
