# Supplementary material for: Mobile App Rating Scale: A New Tool for Assessing the Quality of Health Mobile Apps
Source: JMIR Mhealth Uhealth. 2015 Mar 11;3(1):e27. doi: 10.2196/mhealth.3422 (PMC4376132; doi:10.2196/mhealth.3422)
Supplement: Supplementary file 1 [file mhealth_v3i1e27_app1.pdf]

Papers, publications and materials used for MARS criteria selection

Author / Year	Title	Contains a scale
**Publications**		
Aladwani AM, Palvia PC; 2001[1]	Developing and validating an instrument for measuring user-perceived web quality	Yes
Doherty G, Coyle D, Matthews M; 2010[2]	Design and evaluation guidelines for mental health technologies	No
Eng TR; 2002[3]	eHealth research and evaluation: Challenges and opportunities	No
Finstad K; 2010[4]	The usability metric for user experience	Yes
Handel MJ; 2011[5]	mHealth (Mobile health) – Using apps for health and wellness	No
Ho B, Lee M, Armstrong AW; 2013[6]	Evaluation criteria for mobile teledermatology applications and comparison to major mobile teledermatology applications	No
Kay-Lambkin FJ, White A, Baker AL; 2011[7]	Assessment of function and clinical utility of alcohol and other drug web sites: An observational, qualitative study	Yes
Khoja S, Durrani H et al.; 2013[8]	Conceptual framework for development of comprehensive e-Health evaluation tool	No
Kim P, Eng TR et al.; 1999[9]	Published criteria for evaluating health related web sites: review	No
Lavie T, Tractinsky N; 2004[10]	Assessing dimensions of perceived visual aesthetics of web sites	Yes
Moshagen M, Thielsch M; 2012[11]	A short version of the visual aesthetics of websites inventory	Yes
Oinas-Kukkonen H, Harjumaa M; 2008[12]	A systematic framework for designing and evaluating persuasive systems	No
Olsina L, Rossi G; 2002[13]	Measuring web application quality with WebQEM	No
Schulze K, Krömker H; 2010[14]	A framework to measure user experience of interactive online products	No
Tuch AN, Roth SP, et al.; 2012[15]	Is beautiful really usable? Toward understanding the relation between usability, aesthetics, and affect in HCI	No

**Conference proceedings**		
Law ELC, Roto V et al.; 2009[16]	Understanding, scoping and defining user eXperience: A survey approach	No
Moustakis V, Litos C et al.; 2004[17]	Website quality assessment criteria	Yes
Seethamraju R; 2006[18]	Measurement of user-perceived web quality	Yes
Väätäjä H, Koponen T, Roto V; 2009[19]	Developing practical tools for user experience evaluation – a case from mobile news journalism	Yes
Vermeeren APOS, Law ELC et al.; 2010[20]	User experience evaluation methods: Current state and development needs	Yes
**Manuscripts**		
mHIMSS App Usability Work Group; 2012[21]	Selecting a mobile app: Evaluating the usability of medical applications	Yes
Naumann F, Rolker C; 2000 [22]	Assessment methods for information quality criteria	No
Studies in Health Technology and Informatics; 2013[23]	Issues and considerations for healthcare consumers using mobile applications	No
**Websites**		
Nielsen J; 2003[24]	Usability 101: Introduction to usability	No
www.usabilitynet.org; 2006[25]	What is usability?	No

References

1. Aladwani, A.M. and P.C. Palvia, Developing and validating an instrument for measuring user-perceived web quality. Information & Management, 2002. **39**(6): p. 467-476.
2. Doherty, G., D. Coyle, and M. Matthews, Design and evaluation guidelines for mental health technologies. Interacting with computers, 2010. **22**(4): p. 243-252.
3. Eng, T.R., eHealth research and evaluation: challenges and opportunities. Journal of health communication, 2002. **7**(4): p. 267-272.
4. Finstad, K., The usability metric for user experience. Interacting with Computers, 2010. **22**(5): p. 323-327.
5. Handel, M.J., mHealth (Mobile Health)—Using Apps for Health and Wellness. EXPLORE: The Journal of Science and Healing, 2011. **7**(4): p. 256-261.
6. Ho, B., M. Lee, and A.W. Armstrong, Evaluation Criteria for Mobile Teledermatology Applications and Comparison of Major Mobile Teledermatology Applications. Telemedicine and e-Health, 2013.
7. Kay-Lambkin, F., et al., Assessment of function and clinical utility of alcohol and other drug web sites: An observational, qualitative study. BMC public health, 2011. **11**(1): p. 277.
8. Khoja, S., et al., Conceptual Framework for Development of Comprehensive e-Health Evaluation Tool. TELEMEDICINE and e-HEALTH, 2013. **19**(1): p. 48-53.
9. Kim, P., et al., Published criteria for evaluating health related web sites: review. Bmj, 1999. **318**(7184): p. 647-649.
10. Lavie, T. and N. Tractinsky, Assessing dimensions of perceived visual aesthetics of web sites. International journal of human-computer studies, 2004. **60**(3): p. 269-298.
11. Moshagen, M. and M. Thielsch, A short version of the visual aesthetics of websites inventory. Behaviour & Information Technology, 2013. **32**(12): p. 1305-1311.
12. Oinas-Kukkonen, H. and M. Harjumaa, A systematic framework for designing and evaluating persuasive systems, in Persuasive technology. 2008, Springer. p. 164-176.
13. Olsina, L. and G. Rossi, Measuring Web application quality with WebQEM. Multimedia, IEEE, 2002. **9**(4): p. 20-29.
14. Schulze, K. and H. Krömker. A framework to measure user experience of interactive online products. in Proceedings of the 7th International Conference on Methods and Techniques in Behavioral Research. 2010. ACM.
15. Tuch, A.N., et al., Is beautiful really usable? Toward understanding the relation between usability, aesthetics, and affect in HCI. Computers in Human Behavior, 2012. **28**(5): p. 1596-1607.
16. Law, E.L.-C., et al. Understanding, scoping and defining user experience: a survey approach. in Proceedings of the SIGCHI Conference on Human Factors in Computing Systems. 2009. ACM.
17. Moustakis, V., et al. Website Quality Assessment Criteria. in IQ. 2004.
18. Seethamraju, R., Measurement of User Perceived Web Quality. 2004.
19. Väätäjä, H., T. Koponen, and V. Roto. Developing practical tools for user experience evaluation: a case from mobile news journalism. in European Conference on Cognitive Ergonomics: Designing beyond the Product---Understanding Activity and User Experience in Ubiquitous Environments. 2009. VTT Technical Research Centre of Finland.

20. Vermeeren, A.P., et al. User experience evaluation methods: current state and development needs. in Proceedings of the 6th Nordic Conference on Human-Computer Interaction: Extending Boundaries. 2010. ACM.
21. Health Care Information and Management Systems Society, H., Selecting a Mobile App: Evaluating the Usability of Medical Applications. 2012.
22. Naumann, F. and C. Rolker, Assessment methods for information quality criteria. 2000.
23. Cummings, E., E. Borycki, and E. Roehrer, Issues and considerations for healthcare consumers using mobile applications. Studies in Health Technology and Informatics, 2013. **182**: p. 227-231.
24. Nielsen, J., Usability 101: Introduction to usability. 2003.
25. www.usabilitynet.org. What is usability? http://www.usabilitynet.org/management/b_what.htm.
