# Supplementary material for: Mobile App Rating Scale: A New Tool for Assessing the Quality of Health Mobile Apps
Source: JMIR Mhealth Uhealth. 2015 Mar 11;3(1):e27. doi: 10.2196/mhealth.3422 (PMC4376132; doi:10.2196/mhealth.3422)
Supplement: Supplementary file 3 [file mhealth_v3i1e27_app3.pdf]

Mobile Apps Used for MARS Evaluation

9 Apps used for MARS piloting

Name	Engage-ment	Func-tionality	Aes-thetics	Infor-mation	Sub-jective	Total Mean
Breathe Daily	2.90	4.25	3.00	3.92	2.38	3.52
Meditation Seconds Lite	3.80	3.88	4.50	4.04	3.00	4.05
Interpersonal Dynamics	2.70	4.75	3.50	4.65	2.50	3.90
We Breathe	4.30	4.25	3.17	3.75	2.38	3.87
Everyday Heal with Acupressure	3.70	3.75	3.83	4.08	2.50	3.84
Personal Remedies	2.90	3.50	3.17	3.75	2.63	3.33
Sleep Easily	2.30	4.75	2.33	3.58	1.88	3.24
iThoughtjournal	2.70	4.38	2.83	3.00	2.00	3.23
iPhoria Nature's Music	2.60	4.13	3.83	2.08	2.63	3.16

50 apps used for MARS reliability test

Name	Engage-ment	Func-tionality	Aes-thetics	Infor-mation	Sub-jective	Total Mean
Headspace	4.30	4.50	4.83	4.33	4.25	4.49
Momentum Challenge	4.60	4.75	3.83	4.60	3.25	4.45
NIVEA pure and natural	3.90	4.75	4.67	4.30	3.00	4.4
In Flow- Mood and Emotion	4.20	4.75	4.83	3.46	3.50	4.31
Smiling Mind	3.90	4.50	4.5	4.32	3.75	4.30
Brainy App	4.40	4.50	3.83	4.42	4.13	4.29
PTSD Coach	3.60	4.63	4.33	4.58	3.88	4.29
Healing Time	3.30	4.63	4.17	3.83	2.38	3.98
Moodlytics- The Smart Mood Tracker	3.90	4.25	4.00	3.75	3.00	3.97
Bloom	3.90	4.38	4.33	3.23	2.75	3.96
Humana fit	4.20	3.50	4.17	3.75	3.00	3.90
Kissy Project	2.80	4.75	3.67	4.20	3.13	3.85
Hot Body Yoga	3.00	4.25	3.83	4.00	2.50	3.77
Total Awake	3.90	3.00	4.00	3.58	2.00	3.62
Nursing Psychiatric Drugs	2.10	4.38	3.50	4.38	4.00	3.59
Free Your Mind Hypnosis	2.90	4.38	4.33	2.70	2.00	3.58
Tactical Breather	2.20	4.50	3.50	3.97	1.88	3.54
Thirsty Fish	2.60	4.38	4.17	2.63	1.63	3.44
Deep Relaxation Silvia	2.00	4.38	3.17	4.20	2.25	3.44
Mindful Moments	2.90	4.00	3.00	3.80	1.50	3.43
Stop Stress Pro	3.10	3.88	3.17	3.40	2.38	3.39
Self Help	2.70	3.38	3.33	4.08	3.13	3.37
Conscious	3.00	3.50	3.33	3.60	2.25	3.36
Relax with Andrew Johnson	2.20	4.63	3.33	3.10	2.00	3.31

**Get relaxed free- Personal Hypnosis Program**	1.60	4.63	3.67	3.30	1.75	3.30
**MyPsych**	3.30	3.38	3.17	3.17	1.63	3.25
**ASK and Prevent Suicide**	1.50	4.50	3.00	3.80	2.38	3.20
**EFT Clinic**	1.60	4.13	3.50	3.58	1.50	3.20
**Mindfulness TS**	2.30	3.38	3.33	3.70	1.75	3.18
**Sleep App Insomnia**	2.30	3.75	4.50	2.00	1.63	3.14
**Alpha Mind System**	2.40	3.75	2.83	3.55	1.63	3.13
**Depression Monitor**	2.40	3.63	3.17	3.25	2.00	3.11
**Gratitude Tree**	2.70	4.00	2.67	3.00	2.13	3.09
**Positive Me**	1.80	4.38	3.17	3.00	1.63	3.09
**Mindfulness Meditation- Guided**	1.80	4.38	3.17	2.80	1.38	3.04
**Yoga Fitness Poses**	2.00	3.38	2.67	4.00	1.75	3.01
**Be and it will be**	2.50	4.38	3.33	1.83	1.75	3.01
**Psych Facts**	2.10	3.88	2.83	3.20	1.88	3.00
**Stress Release Hypnosis**	1.70	4.5	3.00	2.60	1.25	2.95
**Mental Workout**	1.60	3.13	4.17	2.80	1.38	2.92
**Animated Quick Reference GAD**	1.90	3.75	3.33	2.48	1.25	2.87
**20 Minute Deep Relaxing Sleep with Hypnosis**	1.90	3.63	2.83	2.98	1.25	2.84
**The Guru With You**	2.20	3.25	3.00	2.83	1.25	2.82
**Binaural Brain Game**	2.90	3.25	2.67	2.37	2.13	2.80
**Aggression Test**	2.50	4.00	2.83	1.67	1.75	2.75
**Won Diary**	2.30	2.75	2.83	2.90	1.38	2.70
**7 Second Meditation- Daily mindfulness**	1.60	4.38	2.00	2.70	1.38	2.67
**Reaction Time by sleep-2-Peak**	2.30	3.50	2.67	2.20	1.63	2.67
**Divine Chants to overcome Fear, Anxiety and Depression**	1.60	3.88	3.33	1.85	1.50	2.66
**Art Helps Healing**	1.50	2.38	2.83	2.17	1.25	2.22
